# Immunotherapy strategies for EGFR-mutated advanced NSCLC after EGFR tyrosine-kinase inhibitors failure

**DOI:** 10.3389/fonc.2023.1265236

**Published:** 2023-10-05

**Authors:** Xingyuan Li, Huayan Huang, Yingjia Sun, Qing Jiang, Yongfeng Yu

**Affiliations:** ^1^ Department of Medical Oncology, Shanghai Chest Hospital, Shanghai Jiao Tong University School of Medicine, Shanghai, China; ^2^ Department of Respiratory and Critical Care Medicine, Fuyang People’s Hospital, Fuyang, China

**Keywords:** non-small cell lung cancer, immunotherapy, EGFR mutation, progression, EGFR-TKIs

## Abstract

**Background:**

This study aimed to investigate the efficacy of immunotherapy, as monotherapy or in combination, comparing to chemotherapy with or without anti-angiogenesis for advanced non-small cell lung cancer (NSCLC) patients progressing to epidermal growth factor receptor (EGFR)-tyrosine kinase inhibitors (TKIs).

**Methods:**

We retrospectively analyzed patients with advanced NSCLC harboring EGFR mutations who received immune checkpoint inhibitors (ICI) and/or chemotherapy after EGFR-TKIs failure at Shanghai Chest Hospital between Aug 2016 and Oct 2022. According to the subsequent immunotherapy regimen, the patients were assigned to ICI monotherapy (IM), IO plus anti-angiogenesis (IA), ICI plus chemotherapy (IC), ICI plus chemotherapy plus anti-angiogenesis (ICA). Eligible patients undergoing standard chemotherapy were assigned to chemotherapy plus anti-angiogenesis (CA) and chemotherapy alone (CM). Efficacy was evaluated according to the RECIST 1.1version, and calculated the objective response rate (ORR) and disease control rate (DCR). Survival curves were plotted using the Kaplan-Meier method, and the median progression-free survival (PFS) was calculated. Differences among survival curves of the six groups were assessed using the log-rank test.

**Results:**

A total of 237 advanced NSCLC patients with EGFR mutations were included in this study. Of the 160 patients who received immunotherapy, 57 received ICI monotherapy, 27 received ICI plus anti-angiogenesis therapy, 43 received ICI plus chemotherapy, and 33 received ICI plus anti-angiogenesis plus chemotherapy. 77 patients received standard chemotherapy, of which 30 received chemotherapy plus anti-angiogenesis and 47 received chemotherapy alone. Patients in ICA group showed significant longer PFS than IM (7.2 vs 1.9 months, *P*=0.011), IA (7.2 vs 4.8 months, *P*=0.009) and CM group (7.2 vs 4.4 months, *P*=0.005). There was no significant difference in PFS between the ICA and IC (7.2 vs 5.6 months, *P*=0.104) or CA (7.2 vs 6.7 months, *P*=0.959) group. Meanwhile, the ICA group showed the highest ORR and DCR (36.4% and 90.9%) compared to the other five groups. The IC group had a higher ORR than the IA and CA group (32.6% vs 7.4% vs 10.0%, respectively), but the DCR was comparable (79.1% vs 74.1% vs 76.7%, respectively). The ORR of the CM group was 6.4% and the DCR was 66.0%. IM group showed the lowest ORR and DCR (1.8% and 36.8%). Treatment-related adverse events (TRAEs) of grade 3 or worse occurred in 9 (27.3%) patients in the ICA group, 6 (20.0%) in the CA group, 7 (14.9%) in the CM group, 5 (11.6%) in the IC group, 5 (8.8%) in the IM group, and 2 (7.4%) in the IA group.

**Conclusion:**

NSCLC patients with positive EGFR mutations after EGFR-TKIs failure received subsequent immunotherapy plus anti-angiogenesis and chemotherapy are likely to have more benefits in ORR, DCR and mPFS.

## Highlights

Key findings

- Immunotherapy plus anti-angiogenesis and chemotherapy may have survival benefits than other regimens in NSCLC patients after EGFR-TKIs failure.

What is known and what is new?

- Patients with advanced NSCLC harboring EGFR mutations have limited benefit from chemotherapy or ICI monotherapy after TKIs failure, and combination therapy is the trend. But the optimal treatment strategy is currently controversial.
- Our study compared the efficacy of four immunotherapy-based therapies with chemotherapy alone or chemotherapy plus anti-angiogenesis to find the optimal treatment regimen for NSCLC patients after EGFR-TKIs failure.

What is the implication, and what should change now?

- Our results can provide references for clinicians' treatment choice. Immunotherapy plus anti-angiogenesis and chemotherapy may be the optimal immunotherapy-based combination therapy, but more prospective studies are needed.

## Introduction

The most common driver gene mutation of non-small cell lung cancer (NSCLC) in East Asians is epidermal growth factor receptor (*EGFR*) mutation (1). EGFR receptor tyrosine kinase inhibitors (TKIs) are preferred for *EGFR*-sensitive mutations in clinical setting; however, most patients experience problems with acquired resistance and disease progression after receiving EGFR-TKIs for approximately 10 to 18 months (2, 3). The use of standard platinum-based dual-drug chemotherapy as subsequent therapy had a limited effect; therefore, more effective treatment strategies should be investigated for patients who progress on EGFR-TKIs therapy.

Immune checkpoint inhibitors (ICI), including cytotoxic T lymphocyte-associated antigens-4 (CTLA-4) inhibitors, programmed cell death 1 (PD-1) inhibitors and programmed cell death ligand 1 (PD-L1) inhibitors, counteract the immunosuppressive effect of tumors and reactivate the immune response of T cells to inhibit tumor cell growth (4). Several studies have demonstrated the efficacy of ICI therapy in advanced NSCLC (5). Immune checkpoint inhibitors such as the PD-1 inhibitor Nivolumab, Pembrolizumab, and the PD-L1 inhibitor Atezolizumab, and the CTLA-4 inhibitor Ipilimumab have been approved for the treatment of advanced NSCLC. However, first-line immunotherapy has poor efficacy for NSCLC with *EGFR* mutations (6). Clinical studies have found that the tumor immune microenvironment is altered after receiving EGFR-TKIs targeted therapy, including an increase in CD8^+^ tumor-infiltrating lymphocytes (TILs) density, TMB, and PD-L1 expression in tumor cells (7, 8). This finding suggests the possibility of ICI monotherapy or ICI-based combination therapy for this population. Results from the IMpower150 and ORIENT31 studies further support the administration of ICI-based combination therapy in this setting (9, 10). However, the phase 3 clinical trials CheckMate-722 and KEYNOTE-789 comparing immunotherapy combined with chemotherapy with chemotherapy alone did not show significant results (11, 12). There remains controversy regarding subsequent therapeutic strategies for NSCLC patients harboring *EGFR* mutations previously treated with EGFR-TKIs. Therefore, our study retrospectively reviewed patients with advanced NSCLC after EGFR-TKIs failure in Shanghai Chest Hospital and analyzed clinical outcomes, safety and relevant influential factors of different treatment options.

## Materials and methods

### Clinical data

This single-center retrospective study was conducted to compare the efficacy of immunotherapy-based regimens with chemotherapy after EGFR-TKIs resistance in *EGFR*-mutant advanced NSCLC patients. Patients with advanced NSCLC after EGFR-TKIs failure at Shanghai Chest Hospital between September 2016 and May 2020 were identified. Patients who interrupted the treatment or did not have imaging data for efficacy assessment were excluded. Due to the small size of the dual immunotherapy patients, they were ultimately not included in the analysis. The baseline clinical characteristics including age, sex, smoking history, tumor pathology type, EGFR mutation subtype, TNM stage, PD-L1 expression status, Eastern Cooperative Oncology Group (ECOG) performance status (PS) score, and lines of therapy were calculated. This study was conducted in accordance with the tenets of the Declaration of Helsinki and the principles of good clinical practice. The protocol and its amendments were approved by the institutional ethical review board of Shanghai Chest Hospital. The ethical review committee waived the requirement for individual informed consent from the patients in this study because of the use of anonymous medical records.

### The inclusion and exclusion criteria

The inclusion criteria were as follows: 1. histologically or cytologically confirmed non-small cell lung cancer; 2. diagnosis of stage IIIB-IV based on the 8th edition of TNM staging system; 3. harboring *EGFR*-activating mutations confirmed by tumor histology, cytology, or circulating tumor DNA; 4. previously received at least one EGFR-TKI and had evidence of radiological disease progression; 5. the participants had an ECOG PS of 0-1; 6. at least one measurable lesion according to the Response Evaluation Criteria in Solid Tumors (RECIST) v1.1.

The exclusion criteria were as follows: 1. Prior systemic immunotherapy before EGFR-TKIs; 2. The clinical data were incomplete; 3. Simultaneous diagnosis of any active autoimmune disease or history of autoimmune diseases; 4. no available imaging data to assess the response to immunotherapy or chemotherapy; 5. discontinuation of treatment for any reason.

### Pathology, biomarkers and molecular diagnostics

Tumor samples collected from biopsy or surgical resection were used for immunohistochemical detection and confirmed pathological diagnosis of NSCLC. The PD-L1 status was determined by immunohistochemistry (IHC) staining. PD-L1 tumor proportion score (TPS) was defined as the proportion of tumor cells with partial or complete cell membrane staining. PD-L1 expression <1% was classified as a negative result; PD-L1 expression ≥1% and <50% was classified as a positive result; PD-L1 expression ≥50% was classified as a strong positive result. EGFR gene subtype mutations were evaluated by detecting blood or tumor tissue samples using next-generation sequencing (NGS) or polymerase chain reaction (PCR).

### Efficacy and safety assessment

Tumor response was assessed using RECIST v1.1. Complete response (CR), complete disappearance of the target lesion, and the short diameter of the pathological lymph nodes decreased to less than 10 mm; partial response (PR), the sum of the target lesion diameter decreased by at least 30%; progressive disease (PD), the sum of the target lesion diameter increased by at least 20% or the appearance of new lesions; stable disease (SD), the increase in diameter of the target lesion did not achieve PD, and the degree of decrease did not achieve PR. Efficacy evaluation includes the overall response rate (ORR) and disease control rate (DCR), where ORR was defined as the proportion of patients who had a CR or PR, and DCR was defined as the proportion of patients who had CR, PR, or SD. Imaging was performed for at least once per cycle during the treatment phase. Interruption of treatment occurred at the time of radiographically identified disease progression. Long term outcomes were evaluated using progression-free survival (PFS), which was measured from the initiation of treatment to radiographic progression. The cut-off date was March 2023, and patients who remained unprogressive at this time were recorded as censored. Adverse events were reported during study treatment and for 30 days after treatment ended and graded as per the National Cancer Institute Common Terminology Criteria for Adverse Events (version 5.0).

### Statistical analysis

Data were analyzed by the software SPSS version 25.0 and GraphPad Prism version 8.0. Continuous variables (e.g age) were presented as medians and ranges. Categorical variables were presented as numbers and percentiles. Survival curves were plotted and median PFS (mPFS) was calculated using the Kaplan-Meier (KM) method. The Log-Rank test was used to compare survival differences between groups. PFS relevant influential factors were explored by evaluating hazard ratios (HR) and associated 95% CIs using the Cox proportional hazard model. Differences in ORR, DCR or the other categorical variables were evaluated using Chi-square (χ2) test or Fisher exact test, as appropriate. Statistical significance was defined as two-sided *P* values of less than 0.05.

## Results

### Patient characteristics

**Figure 1:**
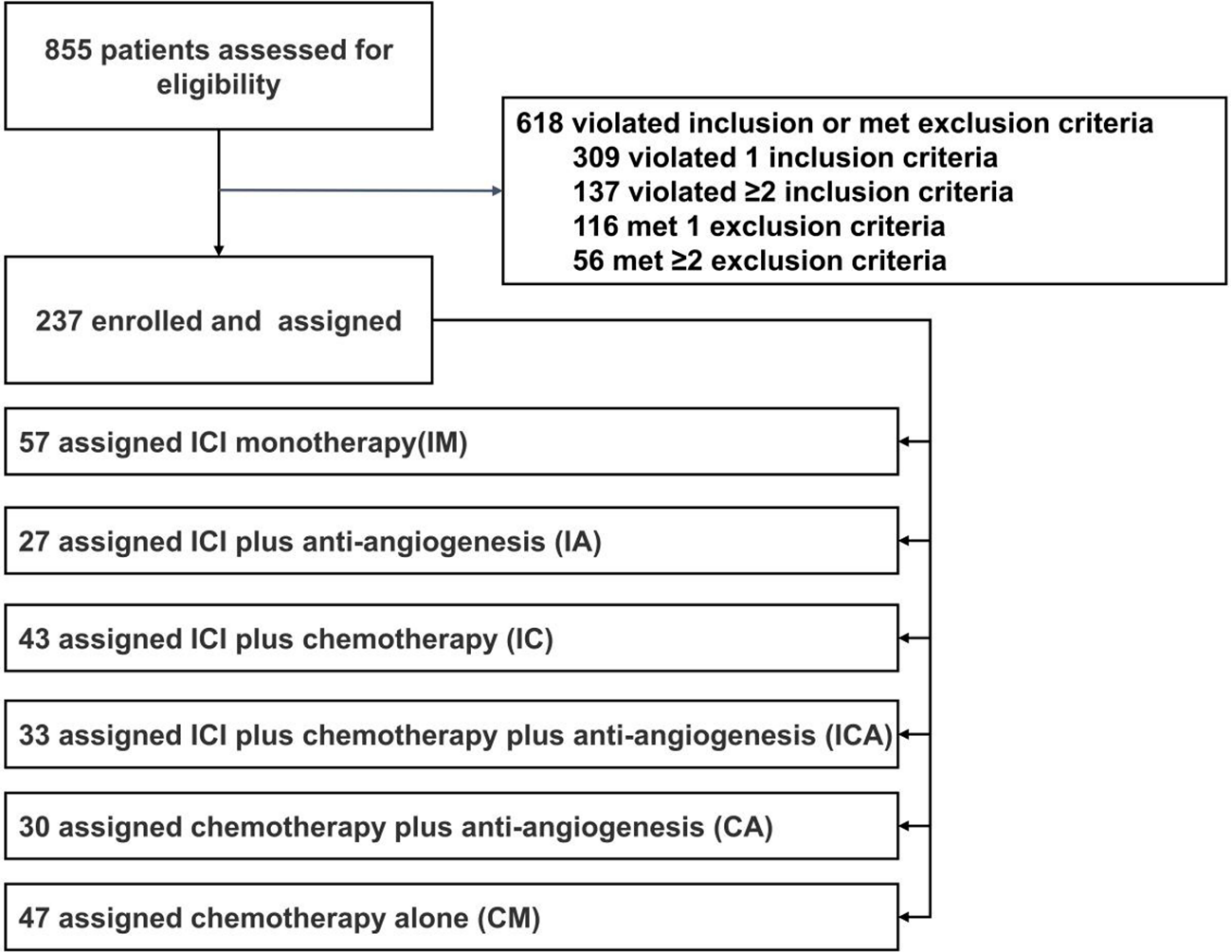
Study flow chart. Between September 2016 and May 2020,855 patients were assessed for eligibility, and 237 patients were finally brought into the analysis, include 57 received ICI monotherapy (IM), 27 received ICI plus anti-angiogenesis (IA), 43 received ICI plus chemotherapy (IC), 33 received ICI plus chemotherapy plus anti-angiogenesis (ICA),30 received chemotherapy plus anti-angiogenesis (CA) and 47 received chemotherapy alone (CM).

In total, 855 patients were assessed for eligibility. Based on the inclusion and exclusion criteria, 237 patients were finally included in the analysis. According to the subsequent regimen, the patients who received immunotherapy were further subdivided into four groups: 57 received ICI monotherapy (IM), 27 received ICI plus anti-angiogenesis (IA), 43 received ICI plus chemotherapy (IC), and 33 received ICI plus chemotherapy plus anti-angiogenesis (ICA). Patients who received standard chemotherapy were separated into two groups: 30 received chemotherapy plus anti-angiogenesis (CA) and 47 received chemotherapy alone (CM) (**Figure 1**). The median age of all patients was 61 years (range 35-77 years), and 56.5% of the patients were female (n=134). 98.7% of patients were diagnosed with lung adenocarcinoma (n=234). Two patients who received immunotherapy were diagnosed with poorly differentiated non-small cell lung cancer. One patient who received chemotherapy was diagnosed with lung squamous cell carcinoma. Of the patients, 89% (n=211) harbored common *EGFR* mutations, with *EGFR* exon 19 deletion (19del) in 54.8% (n=130), *EGFR* exon 21 L858R mutation (21L858R) in 34.2% (n=81), and the remaining 11.0% (n=26) harbored EGFR rare mutations. 66.2% (n=157) of patients were diagnosed with stage IVB, 25.3% (n=60) with stage IVA, and 8.4% (n=20) with stage IIIB or IIIC. All patients had good performance status (ECOG=0-1). 30.8% (n=73) of the patients acquired the T790M mutation after receiving the first or second generation of EGFR-TKIs. PD-L1 expression was detected in 45.6% (n=108) of all patients, and 41.4% (n=98) of those patients had received immunotherapy. In total, 61.6% (n=146) of the patients received PD-1 inhibitors, 5.9% (n=14) received PD-L1 inhibitors, and 32.5% (n=77) received chemotherapy. Detailed treatments of immunotherapy populations are showed in **Table S1**. 36.3% (n=86) of the patients received fourth line or later line of therapy, 29.5% (=70) received third line of therapy, and 34.2% (n=81) received second line of therapy. Except lines of therapy and PD-L1 status, demographic and clinical characteristics were well balanced among the groups at baseline, and the data are listed in **Table 1**.

**Table 1:** Baseline demographic and clinical characteristics of patients stratified by treatment strategies after TKIs failure (n=237).

Characteristics	Total (n=237)	IM (n=57)	IA (n=27)	IC (n=43)	ICA (n=33)	CA (n=30)	CM (n=47)	P value
Age, years, median (range)	61 (35-77)	63 (39-77)	62 (35-73)	58 (35-75)	64 (39-76)	58 (37-74)	61(39-76)	0.157
Gender, n (%)								0.426
Male	103(43.5)	26(45.6)	7(25.9)	20(46.5)	14(42.4)	12(40.0)	24(51.1)	
Female	134(56.5)	31(54.4)	20(74.1)	23(53.5)	19(57.6)	18(60.0)	23(48.9)	
Smoking history, n (%)								0.538
Current or former	53(22.4)	13(22.8)	3(11.1)	12(27.9)	7(21.2)	5(16.7)	13(27.7)	
Never	184(77.6)	44(77.2)	24(88.9)	31(72.1)	26(78.8)	25(83.3)	34(72.3)	
TNM Stage, n (%)								0.713
III Stage	20(8.4)	5(8.8)	3(11.1)	3(7.0)	2(6.1)	2(6.7)	5(10.6)	
IVA Stage	60(25.3)	14(24.6)	3(11.1)	14(32.6)	11(33.3)	9(30.0)	9(19.1)	
IVB Stage	157(66.2)	38(66.7)	21(77.8)	26(60.5)	20(60.6)	19(63.3)	33(70.2)	
PD-L1 percentage expression, n (%)								<0.001
<1%	45(19.0)	6(10.5)	8(29.6)	15(34.9)	13(39.4)	1(3.3)	2(4.3)	
1-49%	33(13.9)	10(17.5)	8(29.6)	4(9.3)	6(18.2)	1(3.3)	4(8.5)	
≥50%	30(12.7)	8(14.0)	3(11.1)	8(18.6)	9(27.3)	0	2(4.3)	
Not examined	129(54.4)	33(57.9)	8(29.6)	16(37.2)	5(15.2)	28(93.3)	39(83.0)	
*EGFR* subtypes, n (%)								*0.810*
19Del	130(54.9)	32(56.1)	18(66.7)	24(55.8)	14(42.4)	18(60.0)	24(51.1)	
L858R	81(34.2)	19(33.3)	7(25.9)	12(27.9)	15(45.5)	10(33.3)	18(38.3)	
Rare mutations^a^	26(11.0)	6(10.5)	2(7.4)	7(16.3)	4(12.1)	2(6.7%)	5(10.6)	
Lines of therapy, n (%)								<0.001
2	81(34.2)	3(5.3)	6(22.2)	15(34.9)	17(51.5)	13(43.3)	27(57.4)	
3	70(29.5)	17(29.8)	3(11.1)	11(25.6)	9(27.3)	14(46.7)	16(34.0)	
≥4	86(36.3)	37(64.9)	18(66.7)	17(39.5)	7(21.2)	3(10.0)	4(8.5)	

^a^Rare mutations included one 18G719X and 18E709K co-mutations, one 18G719X and 20R776C co-mutations, one 19del and 21L861Q co-mutations, one 19del and 4S177L co-mutations, two 20ins, one 20S768I, one 20S768I and 18G719X co-mutations, two 21L858R and 20S768I co-mutations, one 21L858R and 21A871G co-mutations, one 21L858R and 7A289D co-mutations, one 21L858R and 21T854A co-mutations, one 21L858R and 21V834L co-mutations, one 21L858R and 4del and 8S315R co-mutations, two 21L861Q, one 21L861Q and 20V769L co-mutations, one EGFR amplification and 20ins co-mutations in immunotherapy population. One 18G719X and 20S768Ico-mutations, one 18G719X and 241L861Q co-mutations, one 20ins, one 20S768I, one 21L858R and 20S768I co-mutations, one 21L858R and 21L838V co-mutations, one 21L861Q in chemotherapy population. IM, ICI monotherapy; IA, ICI plus anti-angiogenesis; IC, ICI plus chemotherapy; ICA, ICI plus chemotherapy plus anti-angiogenesis; CA, chemotherapy plus anti-angiogenesis; CM, chemotherapy alone; ICI, immune checkpoint inhibitors; TNM, tumor node metastasis classification; PD-L1, programmed cell death ligand 1; EGFR, epidermal growth factor receptor.

### Efficacy evaluation

For the entire study population, the ORR was 14.8% and the DCR was 67.1% (**Table 2**). Comparative analysis of six groups, the highest ORR was observed in the ICA group, followed by the IC, CA, IA, CM group, and the lowest ORR was observed in the IM group (36.4% vs 32.6% vs 10.0% vs 7.4% vs 6.4% vs 1.8%, **Table 2**). Similarly, the highest DCR was observed in the ICA group, followed by the IC, CA, IA, CM group, and the lowest DCR was observed in the IM group (90.9% vs 79.1% vs 76.7% vs 74.1% vs 66.0% vs 36.8%, **Table 2**). The ICA group and IC group showed similar ORR (36.4% and 32.6%, respectively, **Table 2**), which were higher than those of the other four groups. Meanwhile, the ICA group showed a higher DCR (90.9%) than the other five groups (**Table 2**). There was no significant difference between the DCR of the IC, IA, and CA group (79.1% vs 74.1% vs 76.7%, respectively) (**Table 2**). ICI monotherapy resulted in a significant lower DCR (36.8%) than those of other five groups (**Table 2**).

**Table 2:** Efficacy of subsequent therapy of patients.

Efficacy	Total (n=237)	IM (n=57)	IA (n=27)	IC (n=43)	ICA (n=33)	CA (n=30)	CM (n=47)	P value
CR, n (%)	0	0	0	0	0	0	0	<0.001
PR, n (%)	35(14.8)	1(1.8)	2(7.4)	14(32.6)	12(36.4)	3(10.0)	3(6.4)	
SD, n (%)	125(52.7)	20(35.1)	18(66.7)	20(46.5)	18(54.5)	21(70.0)	28(59.6)	
PD, n (%)	77(32.5)	36(63.2)	7(25.9)	9(20.9)	3(9.1)	6(20.0)	16(34.0)	
ORR (%)	14.8	1.8_a_	7.4_a,b_	32.6_b_	36.4_b_	10.0_a,b_	6.4_a_	<0.001
DCR (%)	67.1	36.8_a_	74.1_b_	79.1_b_	90.9_b_	76.7_b_	66.0_b_	<0.001
mPFS (months)	4.9	1.9	4.8	5.6	7.2	6.7	4.4	0.0107

ORR and DCR for each group with the same letter (a or b) are not significantly different. CR, Complete response; PR, partial response; PD, progressive disease; SD, stable disease; ORR, overall response rate; DCR, disease control rate; mPFS, median progression-free survival.

### Long term outcomes

**Figure 2:**
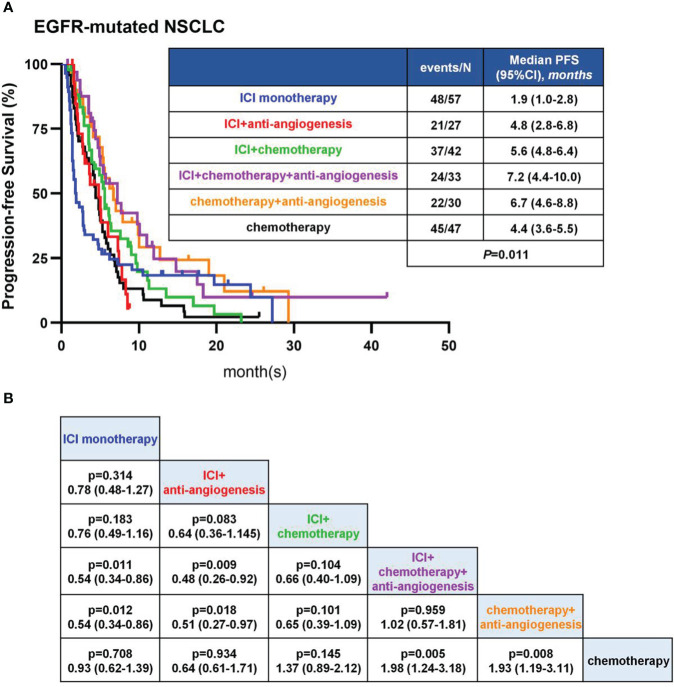
PFS in EGFR-mutant patients. **(A)** Kaplan-Meier analyses in different therapies. **(B)** Pairwise comparisons among therapies in log-rank test.

The median PFS of all eligible patients was 4.9 months (**Table 2**). The mPFS was longest in the ICA group, followed by CA, IC, IA and CM group, and shortest in IM group (7.2 [95%CI: 4.4-10.0 months] vs 6.7 [95%CI: 4.6-8.8 months] vs 5.6 [95%CI: 4.8-6.4 months] vs 4.8 [95%CI: 2.8-6.8 months] vs 4.4 [95%CI: 3.6-5.5 months] vs 1.9 [95%CI: 1.0-2.8 months]) (**Figure 2A**). Patients in ICA group showed significant longer PFS than IM (7.2 vs 1.9 months, *P*=0.011), IA (7.2 vs 4.8 months, *P*=0.009) and CM (7.2 vs 4.4 months, *P*=0.005) group (**Figure 2B**). Similarly, patients in CA group showed significant longer PFS than IM (6.7 vs 1.9 months, *P*=0.012), IA (6.7 vs 4.8 months, *P*=0.018) and CM (6.7 vs 4.4 months, *P*=0.008) group (**Figure 2B**). There was no significant difference in PFS between the ICA and CA (7.2 vs 6.7 months, *P*=0.959) group (**Figure 2B**). The mPFS of IC group was longer than IM (5.6 vs 1.9 months, *P*=0.183), IA (5.6 vs 4.8 months, *P*=0.083) and CM (5.6 vs 4.4 months, P=0.145) group, but without statistical difference (**Figure 2B**). In these analyses, both ICA and CA therapy showed superior mPFS than other therapeutic strategies.

### Safety

TRAEs had the highest incidence rate in the ICA group(26 [78.8%]) followed by the CA group(21 [70.0%]).TRAEs of any grade occurred in a similar proportion of patients in IA, IC, CM group (16 [59.3%] vs 25 [58.1%] vs 30 [63.8%]).While the IM group had the lowest incidence of TRAEs(22 [38.6%]).TRAEs of grade 3 or worse occurred in 9 (27.3%) patients in the ICA group, 6 (20.0%) in the CA group, 7 (14.9%) in the CM group, 5 (11.6%) in the IC group, 5 (8.8%) in the IM group, and 2 (7.4%) in the IA group. The most common TRAEs were leukopenia (4 [7.0%] in the IM group vs 0 in the IA group vs 18 [41.9%] in the IC group vs 19 [57.6%] in the ICA group vs 14 [46.7%] in the CA group vs 21 [44.7%] in the CM group). Details of TRAEs were provided in **Table S2**.

### Clinical factors associated with PFS

A total of 160 patients who received immunotherapy were included in the univariate and multivariate analyses. Age, gender, smoking history, TNM stage, and line of therapy were included as independent variables. Age was taken as a continuous numerical variable, and its measured value was taken. All of the categorical variables were summarized with frequencies and percentages and were analyzed using the χ^2^ or Fisher exact test, as appropriate. Univariate COX regression analysis showed that age, sex, and smoking history were not risk factors for disease progression in the patients receiving immunotherapy (**Table 3**). A more advanced disease stage seems to be related with a shorter PFS (**Table 3**). Patients receiving an earlier line of immunotherapy showed enhanced survival benefits compared with those who received immunotherapy as a later line (**Table 3**). Multivariate COX regression analysis showed that gender, age, smoking history, and TNM stage were not risk factors of disease progression in the immunotherapy group (**Table 3**). Similarly, the earlier immunotherapy was applied, the more it delayed disease progression (**Table 3**). It should be noted that, owing to the limited case size, the univariate and multivariate analyses did not include the *EGFR* mutant subtype and PD-L1 expression status as independent variables.

**Table 3:** Clinical parameters associated with PFS in univariate and multivariate analyses.

Univariate	Age	Gender	Smoking history	TNM stage	Lines of therapy
HR (95%CI)	0.988 (0.971-1.004)	0.971 (0.685-1.378)	1.154 (0.758-1.758)	1.432 (1.066-1.922)	1.382 (1.118-1.710)
p	0.149	0.870	0.505	0.017	0.003
Multivariate					
HR (95%CI)	0.993 (0.976-1.010)	0.826 (0.540-1.263)	1.323 (0.792-2.207)	1.304 (0.957-1.777)	1.309 (1.047-1.635)
p	0.397	0.377	0.285	0.092	0.018

TNM, tumor node metastasis classification; HR, hazard ratios; CI, confidence interval.

### Subgroup analysis of patients with common *EGFR* mutations

**Figure 3:**
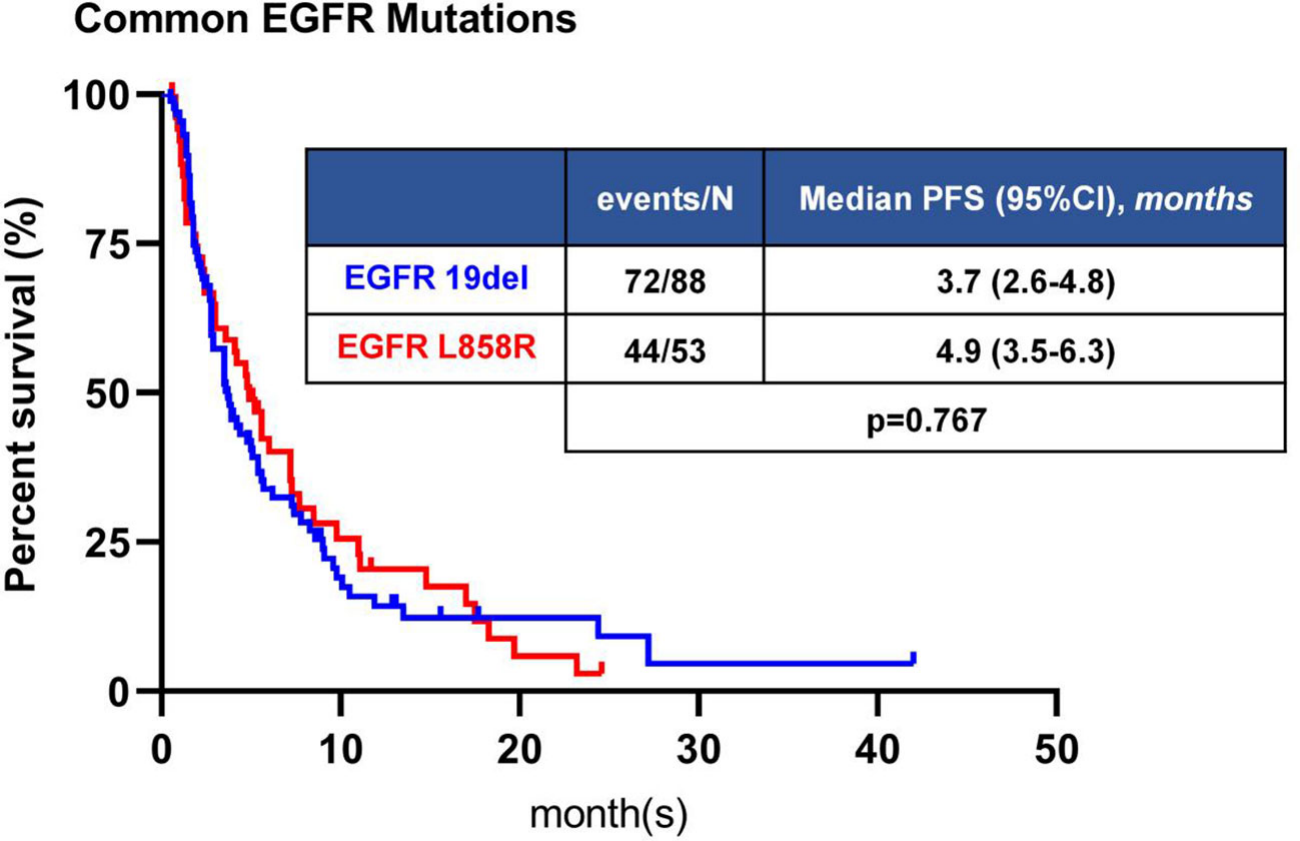
Kaplan-Meier analyses of PFS of patients with common *EGFR* mutation. The difference in PFS was not statistically significant between 19del and L858R.

Among 211 patients with common *EGFR* mutations, 141 patients received immunotherapy, including 88 patients with 19del and 53 patients with 21L858R mutation. There was no significant difference in ORR and DCR between 19del and 21L858R mutations subgroups (*P*=0.066, *P*=0.870, respectively) (**Table S3**). The mPFS was 3.7 months (95%CI: 2.6-4.8 months) in 19del group, and 4.9 months (95%CI: 3.5-6.3 months) in 21L8585R mutations group, with a tendency for longer PFS in 21L858R subgroup, but the difference was not significant (*P*=0.767) (**Figure 3**).

### Subgroup analysis of patients with PD-L1 expression

**Figure 4:**
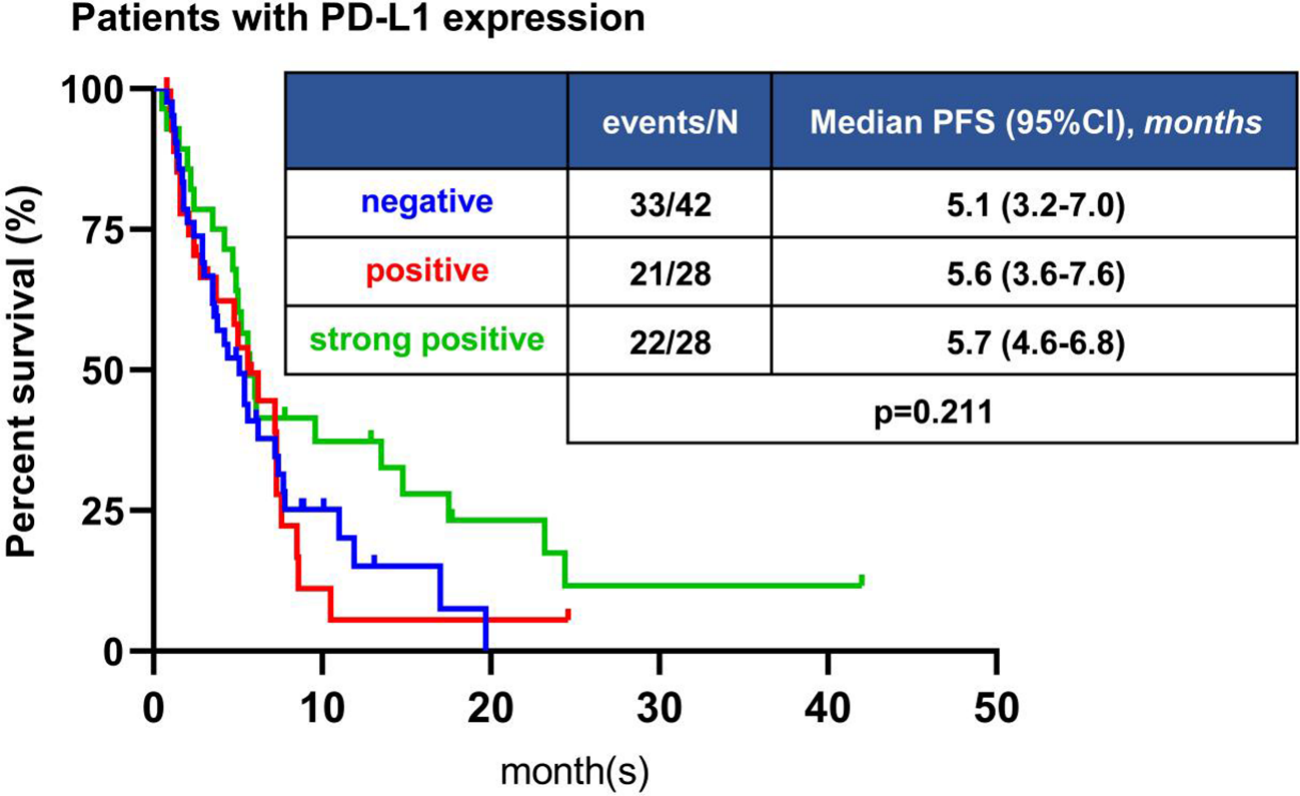
Kaplan-Meier analyses of PFS of patients with different PD-L1 expression. The difference in PFS was not statistically significant among different PD-L1 status.

Among 160 patients receiving immunotherapy, PD-L1 expression was detected in 98 patients, of which 42 patients were PD-L1 negative (<1%), 28 patients were PD-L1 positive (1% -49%), and 28 patients were PD-L1 strongly positive (≥50%). All patients with PD-L1 expression who received immunotherapy had an ORR of 24.5%, DCR of 72.5%, and median PFS of 5.6 months (95%CI, 4.7-6.5 months) (**Table S4**). ORR of PD-L1 negative patients was 21.4%, DCR was 71.4%, median PFS was 5.1 months (95%CI, 3.2-7.0 months); for patients with PD-L1 positive, ORR was 14.3%, DCR was 64.3%, and median PFS was 5.6 months (95%CI, 3.6-7.6 months); for patients with PD-L1 strongly positive, ORR was 39.3%, DCR was 82.1%, and median PFS was 5.7 months (95%CI, 4.6-6.8 months) (**Table S4**). There was no significant difference in the ORR and DCR (*P*=0.078, *P*=0.312, respectively) among these three groups for pairwise comparisons (**Table S4**). The PD-L1 positive and strongly positive groups tended to have a longer PFS than the PD-L1 negative group, but the difference was not significant (*P*=0.211) (**Figure 4**).

## Discussion

The standard first-line treatment for patients with advanced EGFR-mutated NSCLC are EGFR-TKIs. There are limited treatment options for patients who are refractory to third-generation EGFR-TKIs and for T790M-negative patients who have received first- or second-generation EGFR-TKIs. The most commonly recommended treatment is platinum-containing doublet chemotherapy with or without bevacizumab. *In vitro* experiments confirmed that the co-culture system of *EGFR*-mutant tumor cells with immune cells was able to reduce the viability of tumor cells after treatment with PD-1 inhibitors (13). The anti-tumor effect of PD-1 inhibitors has also been demonstrated in preclinical models with *EGFR* mutations (14). These preclinical studies suggested the possibility of treating *EGFR*-mutant NSCLC with ICI. However, the initial clinical findings did not support this. Several prospective studies have shown no survival benefit from the use of immunosuppressive agents versus chemotherapy in NSCLC patients with *EGFR* mutations (15–18). Moreover, most clinical trials of immune checkpoint suppression have also excluded NSCLC patients with driver gene mutations. Therefore, there is an urgent need to develop immune-based combination therapies for NSCLC patients with *EGFR* mutations.

Our study basically included a variety of mainstream and non-mainstream treatment regimens after TKIs resistance. Analysis of these regimens revealed that the mPFS was longest in the ICA group, followed by the CA, IC, IA, and CM group, and the shortest in the IM group. The ICA group had the highest ORR and DCR, followed by the IC, CA, IA, and CM group, and the lowest was in the IM group. Further differential analysis showed that in terms of mPFS, there was no significant difference between the ICA, CA, and IC group for pairwise comparisons. The same results were obtained from the IC, IA, CM, and IM group for pairwise comparisons. There were many interesting conclusions to draw from these analyses.

Combination regimens were superior to monotherapy. In terms of PFS, the ICA group was longer than the CM or IM group in our study. In terms of the ORR and DCR, the ICA group was better than the IM group. The prospective study ORIENT31 included 444 patients with *EGFR* mutations after EGFR-TKIs failure, randomly assigned to sintilimab plus IBI305 plus pemetrexed and cisplatin (ICA), sintilimab plus pemetrexed and cisplatin (IC), or pemetrexed and cisplatin groups (CM). The mPFS of the ICA group was 6.9 months and the mPFS for the CM group was 4.3 months in ORIENT31. The difference in PFS between the two groups was statistically significant (*P*<0.0001) (9). The mPFS in both groups was consistent with the results of our study. Otherwise, our study analysis showed a longer mPFS in the CA group than in the CM or IM group. According to a prospective study, the mPFS of patients with advanced NSCLC received bevacizumab plus chemotherapy was 6.2 months and chemotherapy alone was 4.5 months, and the difference was statistically significant (19). The results were also consistent with the results of our analysis. However, one study showed that chemotherapy combined with anti-angiogenesis was not superior to chemotherapy alone in patients with advanced non-small cell lung cancer (20). Many studies have confirmed the poor efficacy of ICI monotherapy in patients with EGFR-mutant NSCLC. A Japanese randomized controlled study included 102 patients with EGFR-mutant NSCLC after TKIs resistance, with a mPFS of 1.7 months in the nivolumab group and 5.6 months in the carboplatin-pemetrexed group and differences were significant (21). Therefore, CA efficacy was better than IM, which was in line with our expectations.

A subsequent question has to be raised: which regimen has better efficacy comparing ICA with CA? The prospective study Impower150 included 124 chemotherapy-naive NSCLC patients with *EGFR* mutations, of which 91 patients with EGFR sensitive mutations were assigned to three different regimens: atezolizumab plus bevacizumab plus carboplatin plus paclitaxel (ABCP), bevacizumab plus carboplatin plus paclitaxel (BCP), and atezolizumab plus carboplatin plus paclitaxel (ACP). The results showed that in the 124 EGFR mutation subgroup, the HR for ABCP versus BCP was 0.61 (95% CI 0.36-1.03). The mPFS was 10.2 months for the ABCP group, 6.9 months for the BCP group. With respect to the mOS, the ABCP group also improved compared to the BCP group (10). In conclusion, ICA tended to be better than CA, which was consistent with our results. Given that IMpower150 is a small-scale subgroup analysis, it cannot be concluded that ICA was superior to the CA regimen, and the results need to be confirmed by more prospective studies. The mPFS from each treatment group obtained from the IMpower150 study had a gap to compare with our results, and it needed to be considered that we were based on real-world research; most patients received overline therapy, immunotherapy for these patients was a very posterior treatment, and our study showed that the line of therapy on the efficacy of immunotherapy is very obvious. In addition, IMpower150 enrolled patients before osimertinib was approved as first-line treatment.

In addition, pairwise combination regimens of anti-angiogenesis, immunotherapy, and chemotherapy included the CA, IC, and IA group. For the CA or IA group, anti-angiogenesis combination chemotherapy or immunotherapy was feasible. According to previous studies, there was a synergistic effect between chemotherapeutic agents and antiangiogenic agents. Elevated VEGF levels could lead to tumor vascular disorders, increased permeability and interstitial pressure, and affect the delivery of chemotherapeutic agents into the tumor (22). Bevacizumab could promote the delivery of chemotherapeutic agents into the tumor (23). On the other hand, there was also synergy between antiangiogenic agents and immune checkpoint inhibitors, and antiangiogenic agents showed immunomodulatory effects (24), could improve the tumor microenvironment for immunosuppression in patients with EGFR mutations. Our results indicate that the CA group had a significant longer mPFS than the IA group. However, the ORR and DCR were similar between the two groups. For the CA and IC group, combining immunotherapy or anti-angiogenesis based on chemotherapy, the former mPFS was longer than the latter, but there was no significant difference. In the IMpower150 study, in the EGFR mutation subgroup, the HR for PFS with ACP versus BCP was 1.14 (95% CI 0.73-1.18), mPFS 6.9 months in the ACP group, and mPFS 6.9 months in the BCP group. However, in the 56 patients who had previously received EGFR-TKIs, the mPFS of the BCP group was 6.1 months, which was slightly longer than that of the ACP group (mPFS=5.7 months) (10). The results of the subgroup analysis of IMpower150 were similar to our conclusions. Another real-world retrospective study from Shanghai Pulmonary Hospital achieved similar conclusions: the mPFS was 6.90 months in the CA group and 7.59 months in the IC group, and there was no significant difference in PFS (25). However, another retrospective study showed that OS was worse in the IC group comparing with the CA group (HR 2.37, 95%CI 1.09-5.65, *P*=0.030) (26). Similarly, comparing IC with IA regimens, based on immunotherapy combined with chemotherapy or anti-angiogenesis, the former mPFS was longer than the latter, no significant difference was observed in PFS.

Although combination therapy was superior to monotherapy, some exceptions existed. For example, the IC group had a longer mPFS than the CM and IM group, but there was no significant difference in PFS. CheckMate-722 and KEYNOTE-789 study also showed no survival benefit between the IC and CM group (11, 12). However, the results of the latest ORIENT31 second interim analysis showed that mPFS was 5.5 months in the IC group and 4.3 months in the CM group, which was similar with our results. But the difference between these two groups in ORIENT31 trail was statistically significant (*P*=0.016) (27). In addition, the mPFS of the IA group was longer than the CM and IM group, and no significant difference was observed. For IA and CM regimen, the prospective study of Runbo Zhong’s team showed that anlotinib combined with PD-1 inhibitors exhibited better mPFS than chemotherapy group (4.33 vs 3.6 months), a significant increase of nearly 1 month (*P*=0.005). The mOS was 14.17 months in the combination therapy group and 9.00 months in the chemotherapy group, with a significant extension of 5.17 months (*P*=0.029). The DCR of anlotinib combined with PD-1 inhibitor group was 92.1% (28). Compared with our analysis results, we found that the study IA regimen mPFS was longer, the DCR was higher, and the CM regimen mPFS was shorter.

In terms of safety, despite higher rates of grade ≥3 TRAEs, our results show that the ICA group was generally well tolerated with no new safety signals. This is similar to previous studies of such regimens in other patient populations (9, 10).

Multivariate analysis showed that for NSCLC patients with *EGFR* mutations who failed EGFR-TKIs treatment, the earlier they received immunotherapy, the superior benefit in PFS. A clinical study reported that patients who received EGFR-TKIs had alteration in their tumor microenvironment that contributed to immunotherapy. If other treatments were performed during this period, it might lead to the interference of the favorable tumor microenvironment, which could affect the subsequent treatment of ICI (29). Therefore, the timing of immunotherapy administration was equally important.

Subgroups with common *EGFR* mutations receiving immunotherapy were analyzed in our study. The results indicated that patients with the 21L858R mutation tended to have longer mPFS compared with the 19del-mutant population, but the difference was not statistically significant. However, a clinical study showed that patients with the L858R mutation receiving immunotherapy had a better response and an OS benefit compared with 19del population (30).

Analysis of the population detecting PD-L1 status showed that there was no significant difference in the median PFS, ORR and DCR in the PD-L1 high expression group compared with the low expression and negative groups. However, clinically relevant studies were controversial. A single-arm study of toripalimab combined with chemotherapy at the Shanghai Pulmonary Hospital confirmed that the PD-L1 expression levels were not associated with ICI efficacy (31). The study by Shunli Peng et al. found that the ORR in the high PD-L1 expression group was higher than that in the low PD-L1 expression group in *EGFR* mutant patients receiving ICI (32). Another study showed that TMB might be a more suitable biomarker for predicting ICI efficacy than PD-L1 (33). Although high PD-L1 expression suggested a better response to PD-1/PD-L1 inhibitors, it was not absolute, and some patients with high PD-L1 expression still could not benefit from ICI. The reasons included a range of factors, such as tumor mutation burden (TMB), coexisting other gene mutations, microsatellite instability (MSI), “cold” or “hot” tumor microenvironment can affect the efficacy of ICI (34–37).

Our study had several limitations. It was retrospective in nature, leading to the possibility of bias in clinical data. No additional patients receiving dual immunotherapy were included because only eight patients were identified when we reviewed the medical records. However, our study is the first to compare most treatment regimens after EGFR-TKIs failure. Moreover, because driver gene-positive patients did not respond to ICI, most of the current studies did not set an IM group for patients after receiving EGFR-TKIs, and our study precisely included these population. In contrast, our study might able to draw more comprehensive conclusions. To some extent, our analytical results provided a reference for subsequent immunotherapy strategies for similar patients. ICI-based combination therapy, especially immunotherapy combination chemotherapy and anti-angiogenesis might be the preferred treatment regimen for advanced NSCLC patients with *EGFR* mutations after EGFR- TKIs failure.

## Conclusions

In conclusion, our results suggest that immunotherapy combination chemotherapy and anti-angiogenesis are more likely to prolong PFS and improve ORR and DCR than other strategies. However, more prospective clinical studies are still needed to further confirm the efficacy of this strategy.

## Data availability statement

The original contributions presented in the study are included in the article/**Supplementary Material**. Further inquiries can be directed to the corresponding author.

## Ethics statement

The studies involving humans were approved by institutional ethical review board of Shanghai Chest Hospital (ethical approval number:KS22021). The studies were conducted in accordance with the local legislation and institutional requirements. The ethics committee/institutional review board waived the requirement of written informed consent for participation from the participants or the participants’ legal guardians/next of kin because individual consent for this retrospective analysis was waived.

## Author contributions

YY: Conceptualization, Formal Analysis, Methodology, Validation, Writing – review & editing. XL: Conceptualization, Data curation, Formal Analysis, Investigation, Methodology, Software, Validation, Visualization, Writing – original draft, Writing – review & editing. HH: Conceptualization, Data curation, Formal Analysis, Investigation, Methodology, Software, Validation, Visualization, Writing – original draft, Writing – review & editing. YS: Conceptualization, Validation, Writing – review & editing. QJ: Conceptualization, Data curation, Validation, Writing – review & editing.
