# Supplementary material for: Immunotherapy strategies for EGFR-mutated advanced NSCLC after EGFR tyrosine-kinase inhibitors failure
Source: Front Oncol. 2023 Oct 5;13:1265236. doi: 10.3389/fonc.2023.1265236 (PMC10586749; doi:10.3389/fonc.2023.1265236)
Supplement: Supplementary file 1 [file Table_1.docx]

Table S1: ICIs used in immunotherapy populations.

|  | IM group (n=57) | IA group(N=27) | IC group (n=43) | ICA group (n=33) |
| --- | --- | --- | --- | --- |
| Atezolizumab | 8(14.0) | 8(29.6) | 3(7.0) | 1(3.0) |
| Camrelizumab | 2(3.5) | 0(0.0) | 3(7.0) | 0(0.0) |
| Durvalumab | 0(0.0) | 2(7.4) | 0(0.0) | 0(0.0) |
| Geptanolimab | 2(3.5) | 0(0.0) | 0(0.0) | 0(0.0) |
| Nivolumab | 23(40.4) | 2(7.4) | 4(9.3) | 0(0.0) |
| Pembrolizumab | 10(17.5) | 10(37.0) | 16(37.2) | 4(12.1) |
| Sintilimab | 8(14.0) | 1(3.7) | 17(39.5) | 27(81.8) |
| Tislelizumab | 4(7.0) | 4(14.8) | 0(0.0) | 1(3.0) |

Abbreviations: ICIs, immune checkpoint inhibitors; IM, ICI monotherapy; IA, ICI plus anti-angiogenesis; IC, ICI plus chemotherapy; ICA, ICI plus chemotherapy plus anti-angiogenesis; CA, chemotherapy plus anti-angiogenesis; CM, chemotherapy alone.

Table S2: Treatment-related adverse events.

|  | Total (n=237) | | IM (n=57) | | IA (n=27) | | IC (n=43) | | ICA (n=33) | | CA (n=30) | | CM (n=47) | |
| --- | --- | --- | --- | --- | --- | --- | --- | --- | --- | --- | --- | --- | --- | --- |
| Adverse event | All,  n(%) | Grade≥3,  n(%) | All,  n(%) | Grade≥3,  n(%) | All,n(%) | Grade≥3,  n(%) | All,n(%) | Grade≥3,  n(%) | All,n(%) | Grade≥3,  n(%) | All,n(%) | Grade≥3,  n(%) | All,n(%) | Grade≥3,  n(%) |
| Leukopenia | 76(32.1) | 25(10.5) | 4(7.0) | 2(3.5) | 0 | 0 | 18(41.9) | 4(9.3) | 19(57.6) | 8(24.2) | 14(46.7) | 6(20.0) | 21(44.7) | 5(10.6) |
| Anaemia | 4(1.7) | 1(0.4) | 1(1.8) | 1(1.8) | 1(3.7) | 0 | 0 | 0 | 0 | 0 | 0 | 0 | 2(4.3) | 0 |
| Decreased platelet count | 2(0.8) | 2(0.8) | 0 | 0 | 0 | 0 | 0 | 0 | 0 | 0 | 0 | 0 | 2(4.3) | 2(4.3) |
| Nausea | 3(1.3) | 0 | 0 | 0 | 0 | 0 | 0 | 0 | 2(6.1) | 0 | 1(3.3) | 0 | 0 | 0 |
| Vomiting | 6(2.5) | 0 | 2(3.5) | 0 | 1(3.7) | 0 | 1(2.3) | 0 | 1(3.0) | 0 | 0 | 0 | 1(2.1) | 0 |
| Decreased appetite | 7(3.0) | 0 | 1(1.8) | 0 | 1(3.7) | 0 | 0 | 0 | 3(9.1) | 0 | 0 | 0 | 2(4.3) | 0 |
| Diarrhoea | 3(1.3) | 0 | 2(3.5) | 0 | 0 | 0 | 0 | 0 | 1(3.0) | 0 | 0 | 0 | 0 | 0 |
| Constipation | 6(2.5) | 0 | 2(3.5) | 0 | 0 | 0 | 0 | 0 | 3(9.1) | 0 | 1(3.3) | 0 | 0 | 0 |
| Gastrointestinal disorder | 11(4.6) | 0 | 0 | 0 | 0 | 0 | 4(9.3) | 0 | 1(3.0) | 0 | 2(6.7) | 0 | 4(8.5) | 0 |
| Abdominal  discomfort | 3(1.3) | 0 | 1(1.8) | 0 | 1(3.7) | 0 | 0 | 0 | 1(3.0) | 0 | 0(0) | 0 | 0 | 0 |
| Dyspnea | 3(1.3) | 0 | 0 | 0 | 0(0) | 0 | 2(4.7) | 0 | 0 | 0 | 1(3.3) | 0 | 0 | 0 |
| Asthenia | 19(8.0) | 0 | 4(7.0) | 0 | 2(7.4) | 0 | 3(7.0) | 0 | 3(9.1) | 0 | 3(10.0) | 0 | 4(8.5) | 0 |
| Alopecia | 5(2.1) | 0 | 1(1.8) | 0 | 0(0) | 0 | 3(7.0) | 0 | 1(3.0) | 0 | 0(0) | 0 | 0 | 0 |
| Edema | 4(1.7) | 0 | 1(1.8) | 0 | 1(3.7) | 0 | 0 | 0 | 1(3.0) | 0 | 0(0) | 0 | 1(2.1) | 0 |
| Myalgia | 4(1.7) | 0 | 2(3.5) | 0 | 1(3.7) | 0 | 1(2.3) | 0 | 0(0) | 0 | 0(0) | 0 | 0 | 0 |
| Arthralgias | 1(0.4) | 0 | 0(0) | 0 | 0(0) | 0 | 0 | 0 | 1(3.0) | 0 | 0(0) | 0 | 0 | 0 |
| Renal impairment | 4(1.7) | 0 | 1(1.8) | 0 | 0(0) | 0 | 0 | 0 | 2(6.1) | 0 | 1(3.3) | 0 | 0 | 0 |
| Increased aminotransferase | 24(10.1) | 2(0.8) | 4(7.0) | 1(1.8) | 4(14.8) | 1(3.7) | 1(2.3) | 0 | 5(15.1) | 0 | 4(13.3) | 0 | 6(12.8) | 0 |
| Pyrexia | 8(3.4) | 0 | 5(8.8) | 0 | 2(7.4) | 0 | 0 | 0 | 0 | 0 | 0 | 0 | 1(2.1) | 0 |
| Rash | 13(5.5) | 3(1.3) | 3(5.3) | 0 | 3(11.1) | 1(3.7) | 2(4.7) | 1(2.3) | 4(12.1) | 1(3.0) | 1(3.3) | 0 | 0 | 0 |
| Increased amylase | 2(0.8) | 1(0.4) | 1(1.8) | 1(1.8) | 0 | 0 | 0 | 0 | 1(3.0) | 0 | 0 | 0 | 0 | 0 |
| Hypertension | 8(3.4) | 0 | 0 | 0 | 5(18.5) | 0 | 1(2.3) | 0 | 2(6.1) | 0 | 0 | 0 | 0 | 0 |
| Thyroid dysfunction | 3(1.3) | 0 | 0 | 0 | 0 | 0 | 2(4.7) | 0 | 1(3.0) | 0 | 0 | 0 | 0 | 0 |
| Haemoptysis | 5(2.1) | 0 | 1(1.8) | 0 | 3(11.1) | 0 | 0 | 0 | 0 | 0 | 1(3.3) | 0 | 0 | 0 |
| Immune-mediated lung disease | 2(0.8) | 0 | 0 | 0 | 0 | 0 | 1(2.3) | 0 | 1(3.0) | 0 | 0(0) | 0 | 0 | 0 |
| Stomatitis | 12(5.1) | 0 | 2(3.5) | 0 | 5(18.5) | 0 | 1(2.3) | 0 | 3(9.1) | 0 | 1(3.3) | 0 | 0 | 0 |
| Chest pain | 7(3.0) | 0 | 3(5.3) | 0 | 0 | 0 | 2(4.7) | 0 | 1(3.0) | 0 | 1(3.3) | 0 | 0 | 0 |
| Hyperglycaemia | 1(0.4) | 0 | 0 | 0 | 1(3.7) | 0 | 0 | 0 | 0 | 0 | 0 | 0 | 0 | 0 |
| Hand foot syndrome | 2(0.8) | 0 | 0 | 0 | 2(7.4) | 0 | 0 | 0 | 0 | 0 | 0 | 0 | 0 | 0 |
| Epistaxis | 3(1.3) | 0 | 0 | 0 | 1(3.7) | 0 | 1(2.3) | 0 | 0 | 0 | 1(3.3) | 0 | 0 | 0 |

Abbreviations: IM, ICI monotherapy; IA, ICI plus anti-angiogenesis; IC, ICI plus chemotherapy; ICA, ICI plus chemotherapy plus anti-angiogenesis; CA, chemotherapy plus anti-angiogenesis; CM, chemotherapy alone.

**Table S3**: The treatment responses of common *EGFR* mutation types.

|  | CR, n (%) | PR, n (%) | SD, n (%) | PD, n (%) | ORR (%) | DCR (%) | mPFS (months) |
| --- | --- | --- | --- | --- | --- | --- | --- |
| 19del (n=88) | 0 | 11(12.5%) | 45(51.1%) | 32(36.4%) | 12.5% | 63.6% | 3.7 |
| L8585R (n=53) | 0 | 13(24.5) | 20(37.7%) | 20(37.7%) | 24.5 | 62.3% | 4.9 |
| P value | 0.128 | | | | 0.066 | 0.870 | 0.767 |

Abbreviations: CR, Complete response; PR, partial response; PD, progressive disease; SD, stable disease; ORR, overall response rate; DCR, disease control rate; mPFS, median progression-free survival.

**Table S4**: The treatment responses of different PD-L1 expression.

|  | Total  (n=98) | negative  (n=42) | positive  (n=28) | strong positive  (n=28) | P value |
| --- | --- | --- | --- | --- | --- |
| CR, n (%) | 0 | 0 | 0 | 0 | 0.221 |
| PR, n (%) | 24(24.5) | 9(21.4) | 4(14.3) | 11(39.3) |  |
| SD, n (%) | 47(48.0) | 21(50.0) | 14(50.0) | 12(42.9) |  |
| PD, n (%) | 27(27.6) | 12(28.6) | 10(35.7) | 5(17.9) |  |
| ORR (%) | 24.5 | 21.4 | 14.3 | 39.3 | 0.078 |
| DCR (%) | 72.5 | 71.4 | 64.3 | 82.1 | 0.312 |
| mPFS (months) | 5.6 | 5.1 | 5.6 | 5.7 | 0.211 |

Abbreviations: CR, Complete response; PR, partial response; PD, progressive disease; SD, stable disease; ORR, overall response rate; DCR, disease control rate; mPFS, median progression-free survival.
